# Cost of point-of-care lateral flow urine lipoarabinomannan antigen testing in HIV-positive adults in South Africa

**DOI:** 10.5588/ijtld.18.0046

**Published:** 2018-09

**Authors:** R. Mukora, M. Tlali, S. Monkwe, S. Charalambous, A. S. Karat, K. L. Fielding, A. D. Grant, A. Vassall

**Affiliations:** * Aurum Institute, Johannesburg; † School of Public Health, University of the Witwatersrand, Johannesburg, South Africa; ‡ TB Centre, London School of Hygiene & Tropical Medicine, London, UK; § Africa Health Research Institute, School of Nursing and Public Health, University of KwaZulu-Natal, Durban, South Africa

**Keywords:** coinfection, diagnostic tests, point-of-care systems, South Africa, tuberculosis

## Abstract

**INTRODUCTION:**

The World Health Organization recommends point-of-care (POC) lateral flow urine lipoarabinomannan (LF-LAM) for tuberculosis (TB) diagnosis in selected human immunodeficiency virus (HIV) positive people. South Africa had 438 000 new TB episodes in 2016, 58.9% of which were contributed by HIV-positive people. LF-LAM is being considered for scale-up in South Africa.

**METHODS:**

We estimated the costs of using LF-LAM in HIV-positive adults with CD4 counts ⩽ 150 cells/μl enrolled in the TB Fast Track Trial in South Africa. We also estimated costs of POC haemoglobin (Hb), as this was used in the study algorithm. Data on clinic-level (10 intervention clinics) and above-clinic-level costs were collected.

**RESULTS:**

A total of 1307 LF-LAM tests were performed at 10 clinics over 24 months. The mean clinic-level costs were US$12.80 per patient for LF-LAM and POC Hb; LF-LAM costs were US$11.49 per patient. The mean above-clinic-level unit costs for LF-LAM were US$12.06 for clinic preparation, training, coordination and mentoring. The mean total cost of LF-LAM was US$23.55 per patient.

**CONCLUSION:**

At clinic level, the cost of LF-LAM was comparable to other TB diagnostics in South Africa. It is important to consider above-clinic-level costs for POC tests, as these may be required to support roll-out and ensure successful implementation.

## Abstract (FR)

**INTRODUCTION:**

L'Organisation Mondiale de la Santé recommande un diagnostic de la tuberculose (TB) sur place (POC) par dosage de lipoarabinomannane par flux latéral sur l'urine (LF-LAM) chez des personnes sélectionnées positives pour le virus de l'immunodéficience humaine (VIH). L'Afrique du Sud a eu 438 000 nouveaux épisodes de TB en 2016, dont 58,9% étaient des personnes positives au VIH. L'Afrique du Sud envisage une expansion du LF-LAM.

**MÉTHODE:**

Nous avons estimé les coûts de l'utilisation du LF-LAM chez les adultes VIH positifs avec des comptages CD4 ⩽ 150 cellules/μl enrôlés dans l'essai TB Fast Track en Afrique du Sud. Nous avons également estimé les coûts du dosage sur place de l'hémoglobine (Hb), car celui-ci a été utilisé dans l'algorithme de l'étude. Des données relatives aux coûts au niveau des structures de santé (10 centres d'intervention) et au-delà des structures de santé ont été recueillies.

**RÉSULTATS:**

Un total de 1307 tests LF-LAM a été réalisé dans 10 centres en 24 mois. Les coûts moyens pour les centres ont été de US$12,80 par patient pour le LF-LAM et l'Hb sur place ; le coût du LF-LAM a été de US$11,49 par patient. Les coûts unitaires moyens audelà des structures de santé du LF-LAM ont été de US$12,06 pour la préparation, la formation, la coordination et le tutorat. Le coût total moyen du LF-LAM a été de US$23,55 par patient.

**CONCLUSION:**

Au niveau des centres, le coût du LF-LAM a été comparable aux autres diagnostics de TB en Afrique du Sud. Il est important de tenir compte des coûts au-delà des structures de santé des tests sur place, car ils peuvent être nécessaires pour soutenir le lancement et assurer une mise en oeuvre fructueuse.

## Abstract (ES)

**MARCO DE REFERENCIA:**

La Organización Mundial de la Salud recomienda que se utilice la prueba de flujo lateral de lipoarabinomanano en orina (LF-LAM) en el lugar de la consulta (POC) para el diagnóstico de la tuberculosis (TB) en determinadas personas positivas frente al virus de la inmunodeficiencia humana (VIH). En Suráfrica ocurrieron 438 000 nuevos episodios de TB en el 2016, de los cuales el 58,9% afectó a personas positivas frente al VIH; en la actualidad se considera la posibilidad de ampliar la escala de aplicación de la prueba de LF-LAM en el país.

**MÉTODOS:**

Se estimaron los costos de la utilización de la prueba LF-LAM en los adultos positivos frente al VIH con una cifra de linfocitos CD4 ⩽ 150 células/μl que participaron en el estudio ‘***TB Fast Track***’ en Suráfrica. Se estimaron además los costos de determinación de la hemoglobina (Hb) en el POC, pues esta prueba formaba parte del algoritmo del estudio. Se recogieron datos sobre los costos a escala de los consultorios (10 consultorios que participaron en la intervención) y los costos a un nivel superior del consultorio.

**RESULTADOS:**

Se realizaron 1307 pruebas de LF-LAM en 10 consultorios durante 24 meses. El promedio de los costos en los consultorios fue US$12,80 por paciente para la LF-LAM y la Hb en el POC; la prueba LF-LAM costó US$11,49 por paciente. El promedio de los costos unitarios de la prueba FL-LAM en el nivel superior al consultorio fue US$12,06, destinados a la preparación, la capacitación, la coordinación y la tutoría en los consultorios. El promedio del costo total de la prueba LF-LAM fue US$23,55 por paciente.

**CONCLUSIÓN:**

A escala del consultorio, el costo de la prueba LF-LAM fue equivalente al costo de otros medios diagnósticos que se utilizan en Suráfrica. Es importante tener en cuenta los costos de las pruebas aplicables en el POC a un nivel superior del consultorio, pues corresponden a actividades que pueden ser necesarias para respaldar el despliegue de las pruebas y garantizar su utilización eficaz.

TUBERCULOSIS (TB) IS THE LEADING cause of death in human immunodeficiency virus (HIV) positive people. In 2016 alone, TB claimed the lives of 1.67 million people worldwide, 0.37 million of whom were HIV-positive.^1^ Difficulties in the diagnosis of TB among HIV-positive individuals have brought about the need for new diagnostic methods; lateral-flow urine lipoarabinomannan (LF-LAM) antigen testing is one such method.^2^ Recent World Health Organization (WHO) guidelines recommend that countries use LF-LAM to assist in the diagnosis of TB in HIV-positive adult in-patients and out-patients who have signs and symptoms of TB and a CD4 count ⩽ 100 cells/μl.^3^ The WHO also recommends LF-LAM for TB diagnosis in HIV-positive adults who are seriously ill, regardless of CD4 count.^3^

South Africa has one of the world's highest TB incidence rates, estimated at 781 per 100 000 population per year; 59% of individuals with incident TB are HIV-positive.^1^ For countries such as South Africa that are considering scaling up LF-LAM, there is a need to evaluate costs, cost-effectiveness and affordability, given their competing priorities and limited health sector budgets. A previous study evaluating the costs and cost-effectiveness of LF-LAM included only test costs.^4^ To support investment decisions, however, all incremental costs need to be considered in cost-effectiveness analyses. A previous study on the costs of TB diagnostics found that costs during roll-out may be different from those observed at smaller scale.^5^ Measuring and analysing the full costs of implementing TB diagnostics from pilot studies can assist national programmes in making appropriate financial allocations to support the introduction of new TB diagnostic tests.

The present study aimed to estimate costs of LF-LAM used at point of care (POC) in primary health clinics, including those incurred above the clinic level and not directly at the clinics.^6^

## METHODS

### Study setting

The cost estimation was conducted as part of the TB Fast Track Trial, an open, pragmatic, two-arm, cluster-randomised superiority trial with primary health clinics (PHCs) as the unit of randomisation.^7^ The trial evaluated a novel algorithm, which included LF-LAM, which was intended to enable nurses to identify HIV-positive adults with low CD4 counts who were at high risk of TB, and initiate rapid anti-tuberculosis treatment (on the same day or within 1 week of enrolment), followed by prompt initiation of antiretroviral therapy (ART). Clinics were randomised to receive the intervention or standard of care (ratio 1:1). At all clinics, participants who were HIV-positive, with CD4 ⩽ 150 cells/μl, and who had not received ART or TB treatment in the preceding 6 or 3 months, respectively, were enrolled. The trial was conducted between December 2012 and December 2014.

### Intervention description

At enrolment, the research nurse in the intervention clinics performed a POC LF-LAM test and a POC haemoglobin (Hb) test (HemoCue^®^ Hb 201+, HemoCue, Angelholm, Sweden). 7 The POC Hb test was conducted as part of the trial algorithm to assess TB risk. Urine samples were tested using the Alere Determine™ TB LAM Ag assays (Alere Inc, Waltham, MA, USA) according to the manufacturers' guidelines. Stored LF-LAM test strips were removed from the refrigerator to sit at room temperature for at least 1 h before use. A fresh urine sample was then collected from the participant. Using a pipette, 60 μl of the sample was introduced into the LF-LAM test kit and left to incubate at room temperature for 25 min. A timer was set to ensure that the incubation period was accurate and standardised for all samples. At the end of the incubation period, the strips were visually examined with the aid of a desk lamp: the results were interpreted using the reference card provided and recorded on individual worksheets. A positive result required both the control and test band to be visible; positive results were graded by matching the intensity of the test band with bands on the reference card, which ranged from 1+ to 5+.

At the above-clinic level, a research team of six (one project manager, one junior project manager and four study coordinators) supported the clinics with study activities. Their roles involved clinic preparation, training, coordination, quality assurance and mentoring. Clinic preparation included assessing clinics, sourcing equipment, meetings, training clinic-based staff and travel. Training activities included general HIV-TB training, laboratory training for study tests and follow-on training of clinic-based staff during the pilot period. Coordination activities involved provision of required materials and equipment, and organising and chairing team meetings to discuss progress and provide supervision. Mentoring involved supervising the nurses' implementation of the algorithm, as required, to ensure quality test results.

### Scope of costing

We investigated, from a health care system perspective, the full economic costs of LF-LAM as an initial TB diagnostic test in HIV-positive individuals with CD4 count ⩽150 cells/μl in South African PHCs. We also estimated the costs of POC Hb testing conducted as part of the trial, as this was also used in the trial algorithm. We used a micro-costing approach where the resources used and their prices were enumerated.^8^ We costed two time periods: start-up and implementation. We estimated the unit cost per LF-LAM test conducted and the unit cost per POC Hb test conducted.

### Clinic selection

The clinics selected to participate in the trial had the capacity to initiate and deliver TB treatment and ART and had sufficient numbers of patients initiating ART with low CD4 counts.^5^ Clinics with on-site laboratories capable of performing smear microscopy and Xpert^®^ MTB/RIF (Cepheid, Sunnyvale, CA, USA) with same-day results were excluded.^7^ Clinics were also chosen to ensure a combination of both peri-urban and rural PHCs. The sampling frame for this economics study was the 12 intervention clinics in the trial; however, due to practical constraints, costs were collected from 10 clinics. These clinics were in four districts: the Tshwane and Ekurhuleni Districts in Gauteng, the Greater Tubatse District in Limpopo, and the Bojanala District in the Northwest.

### Costing methods

We collected cost data at clinic level and above clinic level from June 2012 to April 2014. At clinic level, all resources were collected using a bottom-up costing approach, where detailed activity and input usage data were found in records (or observations) at the service provider level to estimate unit costs.^5^ Resource use at clinic level was measured using direct observation to ensure that all resource uses were included; any time spent on research activities, such as completing case report forms, was excluded. Resources included were staff salaries, equipment and supplies. To estimate human resource use, the nurses who carried out the investigations were observed performing the diagnostic procedures (POC LF-LAM and POC Hb) 4–8 times at each of the 10 clinics. The resources used and time spent per activity were recorded.

At above clinic level, a top-down approach was used for resource use measurement, where overall expenditures for each input were allocated using an activity-based approach to estimate unit costs.^5^ Recorded activities were grouped into two categories: clinic preparation and training for start-up, and coordination and mentoring during implementation. Human resource time was measured using prospectively completed timesheets, followed by staff interviews for clarification. Research activities were excluded. Financial records from the trial were reviewed to measure other resource use, such as staff transportation and training costs. Results were discussed with project management staff to ensure all research time had been correctly excluded. As coordination and mentoring activities were mostly research-related, only 1% of this cost was included, with the rest allocated to research. We then allocated 60% of the implementation cost to LF-LAM, based on staff reporting of how their time was spent.

For all the abovementioned clinic-level staff costs, two approaches were used to estimate salaries, as staff employed on research projects may cost more than government staff, who are expected to support any LF-LAM introduction in practice. First, payroll costs were converted into a wage per minute by assuming staff worked 8 h a day. Second, National Department of Health (NDoH) salaries were applied to district and provincial coordinators. All other above-clinic-level prices were obtained from project financial records. For supplies, the unit price of the standard package of supplies used at each clinic for LF-LAM and POC Hb testing was sourced from project invoices. Building costs per square metre for a typical public clinic were obtained from NDoH.

We classified any costs that were incurred before LF-LAM was conducted at clinics as start-up costs, and annuitised these over their various useful lives using a discount rate of 3%. The start-up costs were related to capital inputs such as training, buildings, furniture and equipment. A useful life of 2 years was used for training, based on staff turnover, 5 years for furniture and equipment, and 20 years for buildings. All costs are presented in 2014 United States dollars ($US). South African rands (ZAR) were converted into $US using an exchange rate of ZAR10.71 to US$1. Microsoft Excel (MicroSoft, Redmond, WA, USA) was used for all analyses.

### Ethics

The parent trial received approval from the Research Ethics Committees of the University of Witwatersrand, Johannesburg, South Africa (ref: R14/49 M111177); the London School of Hygiene & Tropical Medicine, London, UK (ref: 6099); and the Provincial Research Committees of Gauteng, North West, and Limpopo Provinces, Pretoria, South Africa. The trial was registered with the South African Medicines Control Council (Pretoria, South Africa) as a Phase IV clinical trial (ref N2/19/8/2 #20120157), with the Current Controlled Trials (ref: ISRCTN35344604 (23)), and the South African Registry (ref: DOH-27-0812-3902). All participants provided written informed consent for inclusion in the parent trial; no individual consent was sought for the costing study, as all persons interviewed were those employed by the project.

## RESULTS

Seven peri-urban and three rural clinics were included (Table 1). The mean number of ART initiations per month ranged from 16 to 66 in the 3 months before enrolment. The mean number of LF-LAM tests ranged from 3.71 to 6.63 per month. A total of 1307 LF-LAM tests were performed over the 24 months from December 2012 to December 2014.

**Table 1:**
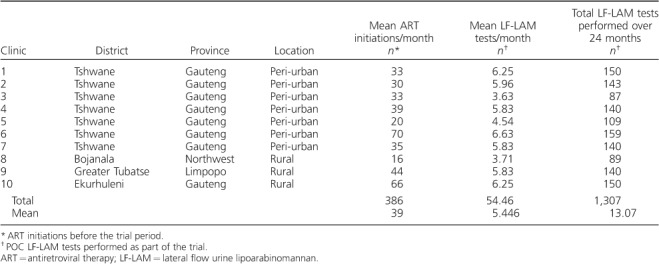
Description of the 10 intervention clinics

The mean clinic-level unit costs were US$12.80 per patient for LF-LAM and POC Hb procedures combined (using non-governmental organisation [NGO] salaries); the mean clinic-level costs of LF-LAM and POC Hb were respectively US$11.50 and US$1.31 (Table 2). For both procedures combined, staff costs (US$8.01) and supplies (US$3.30) were the two largest input costs at clinic level. Staff costs for LF-LAM using research nurse salary scales amounted to US$7.50 per patient, whereas with the NDoH scales they were less than a dollar cheaper, at US$6.86 per patient. Equipment and furniture were the least costly, at US$0.01 per patient.

**Table 2:**
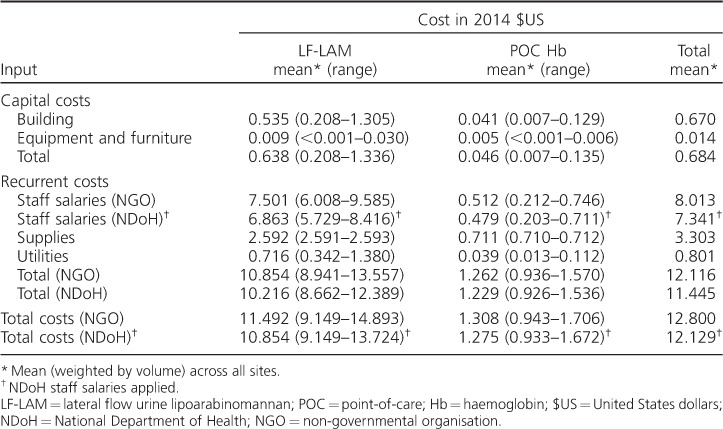
Mean clinic-level unit costs for LF-LAM and POC Hb testing by test and input (2014 $US)

The cost of LF-LAM varied by clinic from US$9.96 to US$13.06 (Table 3), with most of the variation being driven by staff costs. Table 4 presents the total costs incurred above the clinic level over a 24-month period. Building costs here are not annuitized, as they were derived from a monthly cost. The total cost (using NGO salaries) incurred across all 10 clinics was US$15 763. Over half of this cost was incurred during start-up. Most of the funding was spent on training, with the most significant cost driver here being staff salaries. For clinic preparation and mentoring, staff costs were the key cost driver. For coordination, however, equipment and furniture were the main cost drivers; these included laptops, telephones, desks and chairs used above clinic level.

**Table 3:**
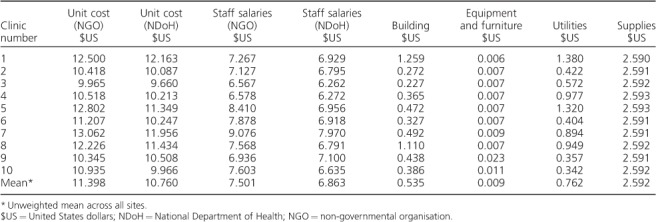
Mean unit costs for lateral flow urine lipoarabinomannan testing by clinic (2014 $US)

**Table 4:**
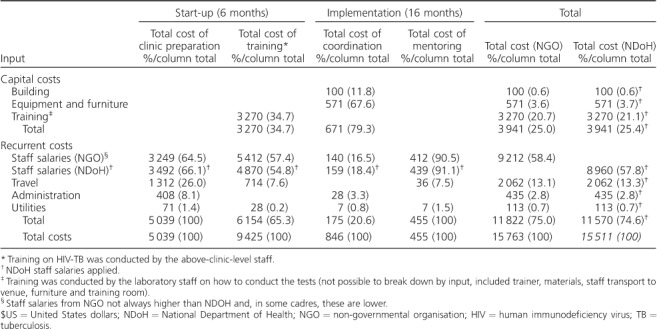
Total costs of lateral flow urine lipoarabinomannan testing at above clinic level by input over a 24-month period, 2014 $US

The above-clinic-level unit costs for LF-LAM were US$12.06 for clinic preparation, training, coordination and mentoring (Table 5). When both above-clinic-level and clinic-level unit costs for LF-LAM were combined, the total cost of LF-LAM per patient was US$23.55 (US$11.49 clinic-level and US$12.06 above-clinic-level). When using NDoH salaries, the total unit cost of LF-LAM was comparable, at US$22.72 per patient (US$10.85 clinic-level and US$11.87 above-clinic-level).

**Table 5:**
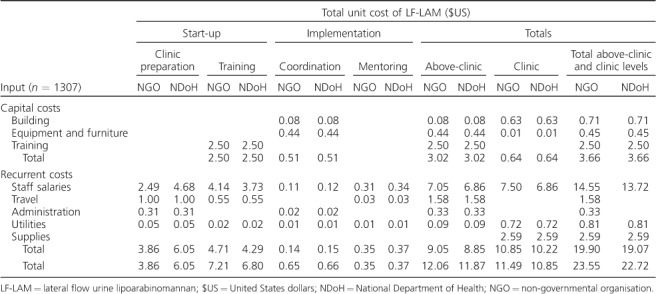
Total LF-LAM unit costs at above clinic and clinic levels by input (2014 $US)

## DISCUSSION

The total unit costs of LF-LAM testing at US$23.55 were considerably higher than previous estimates due to the use of a micro-costing approach and the inclusion of the above-clinic-level costs required to support POC implementation. At both clinic level and above clinic levels, staff costs were the primary cost driver. A previous study considered only the cost of the LF-LAM test kit (US$3.50), thus underestimating clinic-level and above-clinic-level costs by 90%.^3^

The above-clinic-level costs of using LF-LAM were higher than we found for other TB diagnostic tests implemented in South Africa. For example, in a previous study, the above-clinic-level cost of Xpert was found to be US$5.80 per test.^5^ However, as the Xpert study was implemented at the laboratory level and during large-scale roll-out, it benefited from economies of scale. It is also likely that our above-clinic-level costs would fall over time. Nevertheless, it is conceivable that for LF-LAM the ratio of tests to nurses may increase as staff become more proficient at organising and carrying out the tests, leading to a reduction in costs. In addition, if a newer, more sensitive LF-LAM test became available, which could be used more widely, further economies of scale would be realised.

If the LF-LAM test were to be rolled out nationally, some of the activities may also be conducted differently. For example, clinic preparation could be done centrally and would not require multiple site visits. A single trip to conduct regional training for multiple sites on the use of LF-LAM may also suffice. Staff travel costed here was estimated from the head office, 400 km away from the furthest clinic. It is unlikely that support travel costs would need to be based outside the local region for national roll-out. Furthermore, the scale-up of LF-LAM would not require POC Hb. However, national roll-out may incur additional costs to be able to manage the complexity of a technology adoption process at scale. If LF-LAM were implemented only in patients with CD4 count ⩽ 100 cells/μl, then economies of scale would be reduced. It is therefore recommended that LF-LAM be re-costed post roll-out for long-term budget planning.

The study had limitations. First, it should be noted that South Africa has higher staff salaries than other settings, so care should be taken when generalising these costs in settings with lower salaries and higher test volumes. Second, our sampling strategy was driven by the trial's clinic selection; without these restrictions, we might have included better-performing clinics that may have been more efficient (and less costly to support) or, conversely, worse-performing clinics that might have been more costly to support. Third, in the same light, we may have missed some of the health systems investments that may be necessary for weaker clinics; our methods for bottom-up costing, primarily using observation, may also have biased costs downwards, due to the observer, or ‘Hawthorne’, effect. Fourth, the above-clinic-level staff could not be observed performing their duties and we had to rely on their self-completed timesheets; this may have resulted in errors in estimating how much time was spent on specific activities.

## CONCLUSIONS

Our study highlights the importance of conducting comprehensive micro-costing of new TB tests before the decision is taken to roll them out. Accurate, unbiased cost data are essential to inform economic evaluations and financial decisions. For successful implementation, care should be taken to ensure that roll-out is adequately funded, including all the support costs that POC tests often require.
